# Cellular inertia

**DOI:** 10.1038/s41598-021-02384-y

**Published:** 2021-12-10

**Authors:** Ryosuke Ishiwata, Masatomo Iwasa

**Affiliations:** 1 Department of Informatics for Genomic Medicine, Tohoku Medical Megabank Organization, Tohoku University, 2-1 Seiryomachi, Sendai, Miyagi 980-8573 Japan; 2 General Education Center, Aichi Institute of Technology, 1247 Yachigusa, Yakusacho, Toyota, Aichi 474-0392 Japan

**Keywords:** Biophysics, Cell biology, Physics

## Abstract

It has been experimentally reported that chemotactic cells exhibit cellular memory, that is, a tendency to maintain the migration direction despite changes in the chemoattractant gradient. In this study, we analyzed a phenomenological model assuming the presence of cellular inertia, as well as a response time in motility, resulting in the reproduction of the cellular memory observed in the previous experiments. According to the analysis, the cellular motion is described by the superposition of multiple oscillative functions induced by the multiplication of the oscillative polarity and motility. The cellular intertia generates cellular memory by regulating phase differences between those oscillative functions. By applying the theory to the experimental data, the cellular inertia was estimated at $m=3-6$ min. In addition, physiological parameters, such as response time in motility and intracellular processing speed, were also evaluated. The agreement between the experiemental data and theory suggests the possibility of the presence of the response time in motility, which has never been biologically verified and should be explored in the future.

## Introduction

Chemotaxis, defined as directed motion toward the chemoattractant gradient in the surrounding environment, is ubiquitus in biological processes such as wound healing, embryogenesis, neuronal patterning, and tumor dissemination^1–5^. Although many studies have been performed to evaluate static gradients^6,7^, a recent epochal study focused on a dynamic environment; cellular motion was precisely captured under periodic and symmetric traveling waves of the chemoattractant for various wave periods^8^. This study reported a cellular memory, defined as the cellular tendency to maintain their migration direction for some time even after the chemoattractant gradient was reversed. This finding explains why cells can produce a net migration in a specific direction even under symmetric waves, as observed in the aggregation of *Dictyostelium* cells^9^. However, the cause of this cellular memory remains unclear.

In a previous work by the present authors, a phenomenological model was proposed to describe the cellular motion under the traveling wave of the chemoattractant^10^. The model assumed the presence of the sufficient response time from the stimulation to the change in the motility. Due to the phase difference between the oscillative stimulation and motility, the cell speed decreases during the negative gradient of the attractant, and, thus, the net migration under the periodic stimulation was reproduced for various wave periods. However, the experimental results for the instantaneous velocity was not reproduced by the model, in other words, the cellular memory was not explained.

In the present study, we analyzed a model not only incorporating the response time for the motility but also the cellular inertia, which is supposed to induce the motion persistence. As a result, the cellular memory was predicted, as shown that the good agreement between the model solution and the experimental profiles of the instantaneous velocity, as well as the averaged velocity, for three wave periods. Because of the simplicity of the model equation, we can analytically obtain the solution to extrapolate the cellular motion, enabling identification of factors that may contribute for the cellular memory; the cellular motion is composed by the superposition of multiple oscillative functions arising from a combination of oscillating motility and polarity. The cellular inertia governs the phase differences between the oscillative functions, which generates the cellular memory. By applying the theoretical predictions to the experimental results, the cellular inertia was quantified.

## Model

We theoretically investigated the motion of a chemotactic cell migrating in a one-dimensional space. The time evolution of the cellular position *x*(*t*) is assumed to be given by

1 $$\begin{aligned} m{{\ddot{x}}}(t)= & {} -\gamma {\dot{x}}(t)+\chi (t)\nabla S(x(t),t) \end{aligned}$$

where *m* and $\gamma$ represent the cellular inertia and the friction coefficient, respectively. Note that $\gamma$ is set to 1 later, and then both sides of Eq. (1) have the dimensions of the velocity, and *m* has the dimension of time. *S* represents the stimulation by the chemoattractant. Regarding the chemoattractant concentration *c*, the relation $S\sim \log c$ suitably describes the experimental data^10,11^. Corresponding to a previous experimental study^8^, in which the cellular memory was reported, we consider a periodic traveling wave at a constant speed $v_S$; *S*(*x*, *t*) is given by a function of $x+v_S t$. For the motility $\chi$, we incorporate the response time $\tau _m$ from the stimulation by *S* to the change in the motility $\chi$ as performed previously^10^, and set

2 $$\begin{aligned} \chi (t)=\chi _0S(x(t-\tau _{m}),t-\tau _{m}). \end{aligned}$$

As mentioned in the previous study^10^, this relation is inferred from an experimental study in which a certain phase difference was observed between two oscillations of the changes in the chemoattractant concentration and cell speed^12^. In addition, we also assume the linear relation between $\tau _m$ and the wave period *T*, as $\tau _m=:\alpha T+\beta$. This assumption enables to describe the average velocity of cell migration^10^.

By integrating Eq. (1), the instantaneous velocity of the cell is formally expressed as

3 $$\begin{aligned} {\dot{x}}(t)= & {} \frac{\chi _0}{m}\int _0^{t}dt' \Bigl [\mathrm{e}^{-\frac{\gamma }{m}(t-t')}S(x(t'-\tau _m),t'-\tau _m)\nabla S(x(t'), t')\Bigr ]+v(0)\mathrm{e}^{-\frac{\gamma }{m}t}, \end{aligned}$$

where *v*(0) denotes the initial velocity.

## Results

### Instantaneous velocity

To clearly observe the influence of cell inertia, we first consider the stimulation composed of a single sinusoidal wave (see Supplementary Information (SI) A for general periodic functions):

4 $$\begin{aligned} S(x, t)= S_0+S_1\cos (kx+\omega t), \end{aligned}$$

where $|S_0|\ge |S_1|$, $\omega /k=v_S$, and $\omega =2\pi /T$. As the wave speed is much greater than the cell’s speed, the time scale of the change in *S* is much graeter than that of the change in *x*. Therefore, we approximate $x(t-\tau _m)\sim x(t)$ and to be in a constant $x_0$ during the integration. Then, Eq. (3) is reduced to

5 $$\begin{aligned} {\dot{x}}(t)=-\frac{k\chi _0S_0S_1}{\gamma \sqrt{1+\mu ^2}}\sin (kx_0+\omega t-\phi _1)-\frac{k\chi _0S^2_{\mathrm{1}}}{2\gamma \sqrt{1+4\mu ^2}}\sin (2(kx_0+\omega t)-\omega \tau _m-\phi _2)-\frac{k\chi _0S^2_{\mathrm{1}}}{2\gamma }\sin (\omega \tau _m) +A\mathrm{e}^{-\frac{\gamma }{m} t} \end{aligned}$$

where $\mu := m\omega /\gamma$, $\phi _1:=\mathrm{arctan}(\mu )$, $\phi _2:=\mathrm{arctan}(2\mu )$, and *A* represents a constant determined from the initial condition. By neglecting the last term because of the fast damping, the instantaneous cell velocity is approximately given by

6 $$\begin{aligned} {\dot{x}}(t)= -\frac{k\chi _0S_0S_1}{\gamma \sqrt{1+\mu ^2}}\sin (kx_0+\omega t-\phi _1)-\frac{k\chi _0S^2_1}{2\gamma \sqrt{1+4\mu ^2}}\sin (2(kx_0+\omega t)-\omega \tau _m-\phi _2)-\frac{k\chi _0S^2_1}{2\gamma }\sin (\omega \tau _m). \end{aligned}$$

**Figure 1:**
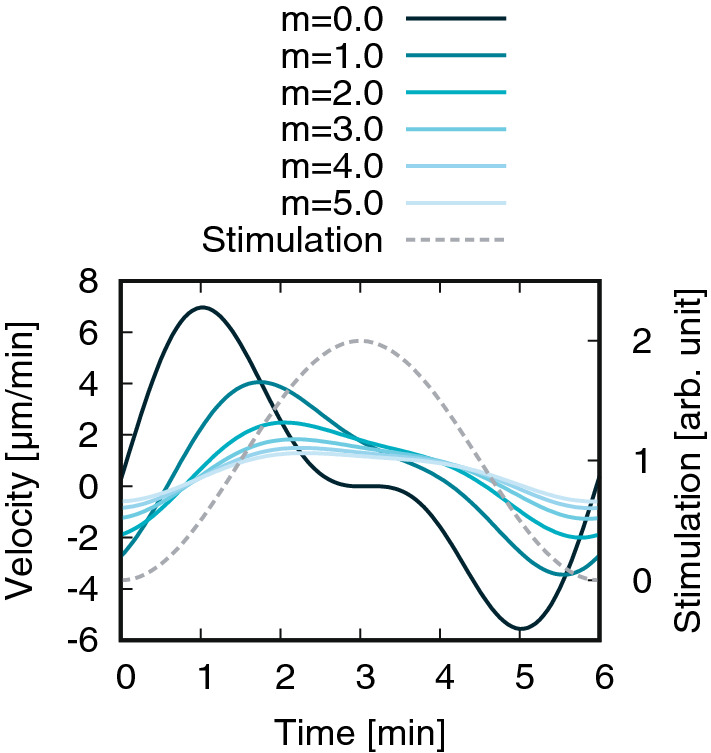
Dependence of the instantaneous velocity, Eq. (6), on the cellular inertia *m* $[\mathrm{min}]$. Other parameters are $T=6$ min, $\alpha =0.5$, $\beta =0.3\ \mathrm{min}$, $S_0=1.0$ $S_1=-1.0$, $\gamma =1$, $\chi _0=1000$, and $\lambda (=2\pi /k)=1300\mu m$.

Figure 1 shows this approximate velocity (6) for various values of inertia *m*. This approximate solution favorably corresponds to the numerical solution obtained using Eq. (1) (Fig. S1a in SI).

We can see from Eq. (6) that the velocity of the cell consists of not only the primary periodic function of the period of *T* (the first term) but also of the less amplified secondary one with the half period (the second term) and a constant (the third term). These qualitatively different factors are generated from the multiplication of the two oscillating quantities with the period of *T*, motility $\chi$, and polarity $\nabla S$. Noteworthy, this characteristic is independent of the presence or absence of the inertia.

### Averaged velocity

Only the constant third term contributes to the averaged velocity, ${\bar{v}}:=\int _t^{t+T}{\dot{x}}(t')dt'$, namely,

7 $$\begin{aligned} {\bar{v}}= & {} -\frac{k\chi _0S^2_1}{2\gamma }\sin (\omega \tau _m). \end{aligned}$$

Since this expression does not include $\mu$, the averaged velocity does not depend on the cellular inertia. In Fig. S1b in SI, this analytical expression compares favorably to the numerical results.

### Influence of intertia

Let us see the influence of the cellular inertia on the cellular motion. In the case of the absence of the cellular inertia, i.e., $m=0$, as we can see from Fig. 1 and exactly see from Eq. (6), the cell velocity is always negative when the stimulation gradient is negative. Therefore, if the cellular inertia is not incorporated, the cellular memory is not produced. Concerning the relation between inertia and velocity, we can find three characteristics from Eq. (6); as the inertia increases, 1) the amplitude of the two periodic components decreases, 2) the phase delay from the stimulation to the two periodic responses, $\phi _1$ and $\phi _2$, increases, and 3) the phase difference between the primary and secondary periodic functions, $\phi _2-\phi _1$, increases. Thus, mainly due to the characteristic 3), the tail after the primary peak becomes longer with increasing inertia. This tail leads to positive velocity even under negative gradient of the stimulation, namely, cellular memory.

**Figure 2:**
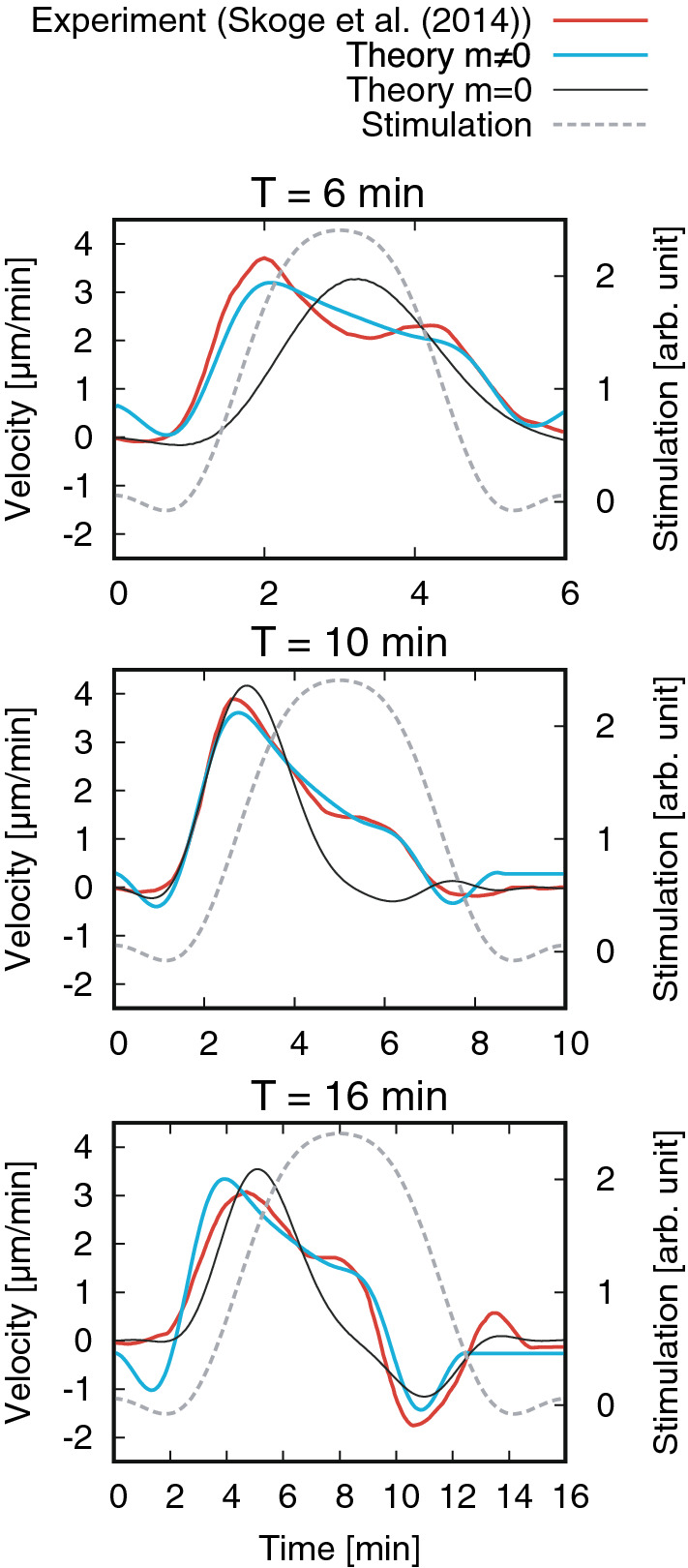
Instantaneous velocities obtained experimentally^8^ and theoretically. Parameter values are ($T=6\ \mathrm{min}$) $m=4\ \mathrm{min}$, $\alpha =0.5$, $\beta =0.2\ \mathrm{min}$, $\chi _0=1639$, and $\sigma =0.94$; ($T=10\ \mathrm{min}$) $m=3\ \mathrm{min}$, $\alpha =0.5$, $\beta =0.5\ \mathrm{min}$, $\chi _0=743$, and $\sigma =1.16$; and ($T=16\ \mathrm{min}$) $m=6\ \mathrm{min}$, $\alpha =0.5$, $\beta =0.3\ \mathrm{min}$, $\chi _0=1137$, and $\sigma =1.29$. For the results of $m=0$, ($T=6\ \mathrm{min}$) $\alpha =0.7$, $\beta =0.7\ \mathrm{min}$, $\chi _0=295$, and $\sigma =0.50$; ($T=10\ \mathrm{min}$) $\alpha =0.4$, $\beta =4.1\ \mathrm{min}$, $\chi _0=201$, and $\sigma =0.93$; and ($T=16\ \mathrm{min}$) $\alpha =0.8$, $\beta =1.7\ \mathrm{min}$, $\chi _0=200$, and $\sigma =0.91$. Reproduced from Skoge et al. PNAS **111**: 14448 (2014), all rights reserved.

## Discussion

### Comparison with experiments

In the experiment analyzing *Dictyosteliym* cells^8^, the instantaneous and average velocities are measured by observing cell migration driven by periodic and symmetric traveling waves of the chemoattractant with varying wave periods. Corresponding to this experimental study, we next set *S* to

8 $$\begin{aligned} S(x,t)=\sum _{j=0}^{3}S_j \cos (j(kx+\omega t)), \end{aligned}$$

where $S_0=1.000$, $S_1=-1.346$, $S_2=0.233$ $S_3=0.172$ (Fig. S2 in SI), $\lambda :=2\pi /k=1300{\mu m}$, and $T=6, 10\ \text {or}\ 16 \text {min}$.

Figure 2 shows the instantaneous velocities obtained experimentally^8^ and theoretically. For each wave period, the presented theoretical result shows the best fit to the experimental result among various combinations of parameter values, with the introduction of a scaling parameter $\sigma$ in the direction of time (see SI C for the detailed procedure used for the comparison with the experimental data). For all of the three wave periods, the shapes of the graphs are similar; long-tails after the primary peak are produced when the inertia is considered (i.e., $m\ne 0$) whereas are not produced when inertia is neglected (i.e., $m=0$). Table 1 shows the parameters estimated from this comparison.

**Figure 3:**
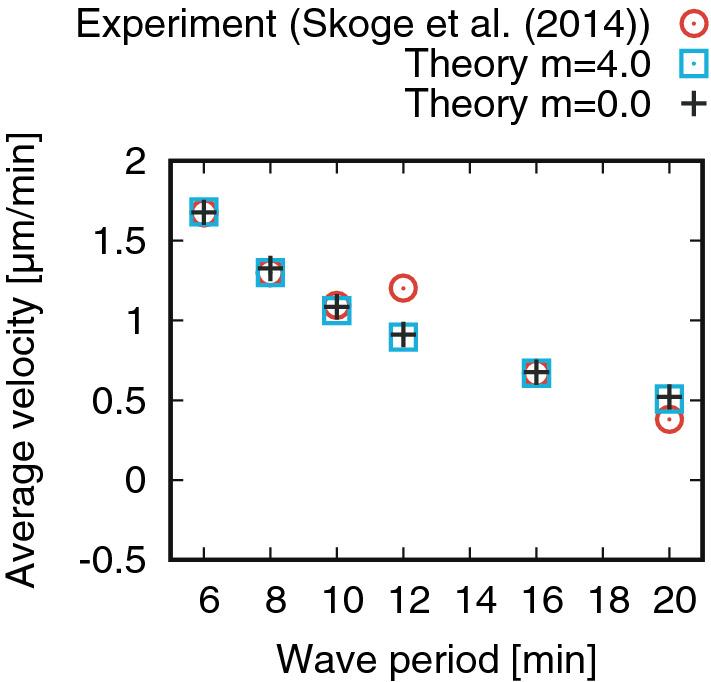
Comparison of the average velocity between the experimental^8^ and theoretical values. Parameter values are ($m=4.0\ \mathrm{min}$) $\alpha =0.5$, $\beta =0.2\ \mathrm{min}$, and $\chi _0=1639$; and ($m=0.0\ \mathrm{min}$) $\alpha =0.5$, $\beta =0.6 \ \mathrm{min}$, and $\chi _0=663$. Reproduced from Skoge et al. PNAS **111**: 14448 (2014), all rights reserved.

Figure 3 shows the average velocities obtained experimentally^8^ and theoretically. The presented theoretical result shows the best fit to the experimental result among various combinations of parameter values (see SI C for the detailed procedure used for comparison with experiments). From the comparison, the parameters, $\alpha$ and $\beta$, are estimated at $\alpha =0.5$, $\beta =0.4-0.8$ (Fig. S3 in SI). These values overlap with those estimated from the instantaneous velocity (Table 1). Notably, as analytically predicted above, the average velocity is well reproduced if intertia is not incorporated.

### Estimation of cellular inertia

According to the results, cellular inertia is estimated as $m=3-6\ {{\mathrm{min}}}$. Thus, the relaxation time for the cellular migration velocity is 3-$6\, {{\mathrm{min}}}$. The molecular basis of this relaxation process should be further explored in dynamic environments in future studies. As the value of $\gamma$ has been shown to be $\gamma =0.07 \,\mu \text {N min}/\mu \text {m}$ in a previous study^13^, the “mass” of the cell is estimated on the order of tons in SI units. While *Dictyostelium* cells have been often assumed to move in an overdamped manner, this result suggests that the inertia cannot be neglected to capture the precise cellular motion pattern, and to explain the cellular memory.

### Consistency with other studies

When the spontaneous random migration is modelled using the Langevin equation, the cellular inertia, also called persistence time, can be calculated from the cellular trajectories^14^. According to previous experiments using *Dictyostelium* cells, persistence times were estimated at $0.72\mathrm{min}$^15^, $3.4\mathrm{min}$^16^, $3.8\mathrm{min}$^17^, and $8.8\mathrm{min}$^18^. Herein, the identified $m=3-6$ min, is within the range of these results.

**Table 1:** Parameter values used or estimated in this study.

	Symbol	Description	Value	Unit	Source
Cellular properties	*m*	Inertia	3 – 6	$\min$	Estimated in this study.
	$\chi _{0}$	Motility magnitude	7–$16\times 10^2$	$\mu \text {m}^2/\min$	
	$\alpha$	Response time in motility $\tau _m=:\alpha T+\beta$	0.5	–	
	$\beta$		0.2–0.5	$\min$	
	$\sigma$	Processing speed factor	0.94–1.29	–	
	$\gamma$	Friction coefficient	1	–	Arbitrarily settable.
Stimulation properties	$T(=2\pi /\omega )$	Wave period	6, 10, 16	$\min$	Set from Skoge et al. (2014).
	$\lambda (=2\pi /k)$	Wave length	1300	$\mu \text {m}$	
	$S_{0}$	Fourier coefficient	1.000	–	
	$S_{1}$		$-1.346$	–	
	$S_{2}$		0.233	–	
	$S_{3}$		0.172	–	

### Response time in motility

A previous study^10^, considering the motility response time, $\tau _m$, accurately explained the relation between the average velocity and wave period. In the present study, this consideration also accurately describes the cell motion, not only regarding the average velocity but also the instantaneous velocity. As the magnitude of $\beta$ is relatively small, the relation between the response time and wave period is approximately estimated at $\tau _m\sim 0.5T$. In other words, when we compare the oscillations of the stimulation *S* and motility $\chi$, their phases were almost in anti-phase. The biochemical origin of this phase difference remains unclear.

### Information processing speed

The scaling parameter $\sigma$ in the direction of time may be physiologically interpreted as the processing speed of the intracellular signal transduction relative to the stimulation; this value indicates the acceleration or deacceleration when $\sigma >1$ or $<1$, respectively. Since the introduction of $\sigma$ markedly improves the correspondence between the theoretical and experimental results (Fig. S6), the processing speed may vary depending on the stimulation period. These results suggest that the processing may be accelerated as the stimulation period increases, since $\sigma =0.94, 1.16, 1.29$ when $T=6, 10, 16$ min, respectively.

## Conclusion

We investigated a mathematical model describing the cell migration to understand why the cellular motion exhibits memory, which is the persistent motion maintained by a cell even after experiencing a chemoattractant gradient reversal^8^. The model incorporates two time scales: persistence time induced by the cellular inertia and motility response time. According to the analysis, the solution of the model shows cellular memory, in which cellular motion is described by superposition of multiple oscillations which have different periods originating from the multiplication of two oscillative quantities: the motility $\chi$ and the polarity $\nabla S$. The cellular inertia determines the temporal length of the memory (Fig. 1), namely, the peak-to-peak distance between those oscillations.

Correspondence with the instantaneous velocity experimetally observed was markedly improved compared with the model with no cellular inertia (Fig. 2), without loss of consisitency in the average velocity (Fig. 3). Parameters characterizing the cellular properties including the cellular inertia were evaluated (Table 1). The magnitude of the cellular inertia was comparable to that observed for random cell migration. This consistency suggests that velocity relaxation in chemotactic migration and the persistence in spontaneous migration can be understood within the same framework; this may help the discovery of a unified intracellular mechanism underlying these different types of migration in future studies.

In the future, the model should be experimentally verified. One of the key assumptions of the model is the presence of significant response time in motility $\chi$ (or is mathematically equivalent to the negative correlation with the stimulation). Although this assumption was inferred from an experimental study^12^, biological basis of this assumption is quite unclear and should be explored. While $\nabla S$ has the directional feature determining the migration direction, the motility $\chi$ represents a non-directional quantity determining the cell speed. Therefore, it may be required to investigate the factors controlling cell speed, without limiting to those involved in the directional sensing mainly studied in^8^. For example, the cell-substrate adhesion may be a potential candidate since, according to the physics (Newton’s second law), cell speed is regulated by the applied force through changes in the acceleration. For a cell moving on a substrate, the force applied from its environment is the adhesive force. In fact, it has been reported that the cellular adhesion would influence on the cellular migratory activity^19,20^. Thus, it is worth investigating the adhesion and/or the molecules involved in such mechanism under a dynamic environment as set in previous works^8,12^.

## Supplementary information

Supplementary Information.
