# Supplementary material for: Cellular inertia
Source: Sci Rep. 2021 Dec 10;11:23799. doi: 10.1038/s41598-021-02384-y (PMC8664931; doi:10.1038/s41598-021-02384-y)
Supplement: Supplementary file 1 — Supplementary Information. [file 41598_2021_2384_MOESM1_ESM.pdf]

Supplemental Information

Ryosuke Ishiwata[1] and Masatomo Iwasa[2]

[1]Department of Informatics for Genomic Medicine, Tohoku Medical Megabank Organization, Tohoku University, 2-1 Seiryomachi, Sendai, Miyagi 980-8573, Japan
[2]General Education Center, Aichi Institute of Technology, 1247 Yachigusa, Yakusacho, Toyota, Aichi 474-0392, Japan

ABSTRACT

This document provides the Supplementary Information of the paper entitled ”Cellular Inertia“.

A: Solution for multiple wave component.

We consider a wave of the stimulation S propagating at a constant speed:

$$S(x,t) = \sum_{j=0}^{\infty} S_j \cos(j(kx+\omega t)), \tag{S1}$$

with constants $\{S_j\}_{j=0,1,2,...}$. With the same approximation considered in the main text, we obtain the instantaneous velocity $\dot{x}(t)$ as

$$\begin{aligned}\dot{x}(t) &= \frac{\chi_0 k}{\gamma}\Bigg\{-\sum_{l=1}^{\infty}\frac{lS_0S_l}{\sqrt{1+l^2\mu^2}}\sin(l(kx_0+\omega t)-\phi_l)-\sum_{j=1}^{\infty}\sum_{l=1}^{\infty}\frac{lS_jS_l}{2\sqrt{1+(j+l)^2\mu^2}}\sin((j+l)(kx+\omega t)-j\omega\tau_m-\phi_{jl}^{+}) \\ &\quad +\sum_{j\neq l,j,l\geq 1}^{\infty}\frac{lS_jS_l}{2\sqrt{1+(j-l)^2\mu^2}}\sin((j-l)(kx+\omega t)-j\omega\tau_m-\phi_{jl}^{-})-\sum_{j=1}^{\infty}\frac{jS_j^2}{2}\sin(j\omega\tau_m)\Bigg\},\end{aligned} \tag{S2}$$

where $\phi_l = \arctan(l\mu)$, $\phi_{jl}^{+} = \arctan((j+l)\mu)$, $\phi_{jl}^{-} = \arctan((j-l)\mu)$. The average velocity $\bar{v} := \int_t^{t+T}\dot{x}(t')dt'$ is given by

$$\bar{v} = -\sum_{j=1}^{\infty}\frac{k\chi_0}{2\gamma} jS_j^2\sin(j\omega\tau_m), \tag{S3}$$

which does not depend on the cellular inertia m.

B: Comparison between analytical and numerical solution.

Fig. S1 shows the comparison of the analytical results Eq. (6) in the main text and the numerical solution obtained using the model equation Eq. (1). Fig. S1a and S1b show the profile of the instantaneous velocity and the average velocity, respectively. The case for $m = 0$ is also shown for reference.

C: Procedure for comparing experimental and theoretical data

Comparison bewteen the theory and experiment was performed as follows: For the wave form of the stimulation, the logarithm of the cAMP concentration is set to be a quadratic function as shown in Fig. S2 in the experiment Ref. 8 in the main text. This form is not suitable for applying the theory because of the divergence of the derivative at the edges of the quadratic. We approximate the function by a sum of sinusoidal functions up to the third order:

$$S(x,t) = \sum_{j=0}^{3} S_j \cos(j(kx+\omega t)), \tag{S4}$$

where $S_0 = 1.000$, $S_1 = -1.346$, $S_2 = 0.232$ $S_3 = 0.172$. Fig. S2 also shows this profile. Corresponding to the experiment Ref. 8, $\lambda(:= 2\pi/k)$ is fixed at 1300 μm^{-1} and $T(:= 2\pi/\omega)$ varies 6, 10, and 16 min.

The theoretical instantaneous velocity, $\dot{x}_{theor}(t)$, is calculated by numerically solving Eq. (1) according to the algorithm provided in[S1]. The time series of $\dot{x}_{theor}(t)$ is obtained after 10 wave periods have passed to eliminate the effect of the initial

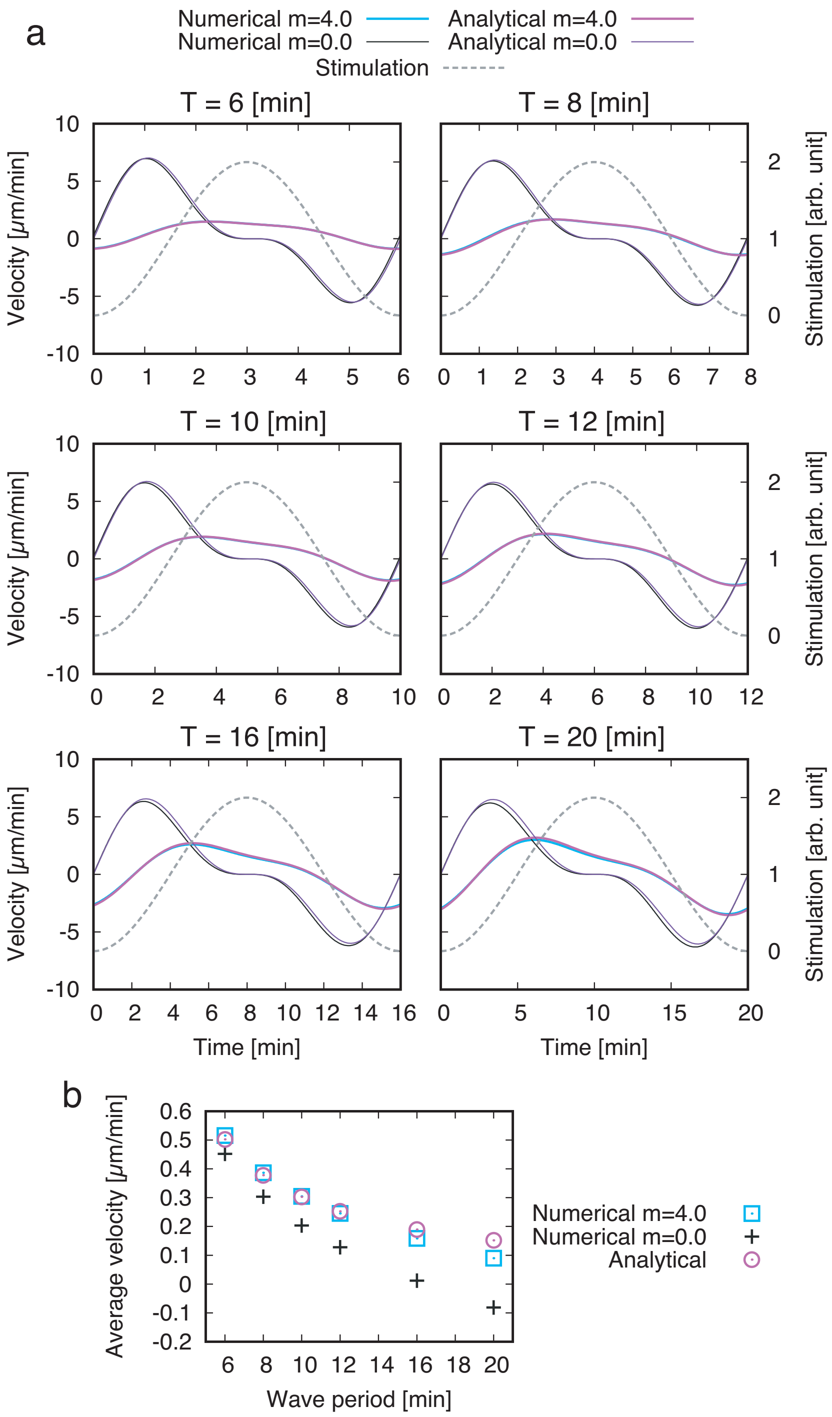

Figure S1. (a) Numerical solutions of the model equation Eq. (1) and the analytical approximate solution Eq. (6) for $m = 0.0$ and 4.0 min and for $T = 6$, 8, 10, 12, 16 and 20 min when the waveform is a single sinusoidal Eq. (4), and (b) the relation between the average velocity and the wave period. Parameter values are $\gamma = 1$, $\alpha = 0.5$, $\beta = 0.2$ min, $S_0 = 1.0$, $S_1 = -1.0$ and $\chi_0 = 1000$.

condition, and capture the periodic behavior. Parameter values are taken from $0 \leq m \leq 10$ min at every 1 min, from $0.0 \leq \alpha \leq 1.0$ at every 0.1, and from $0.0 \leq \beta \leq 5.0$ min at every 0.1 min. γ is fixed at 1 without the loss of generality. Experimental data are extracted by manually digitizing Fig.1(e) in Ref. 8 with ImageJ software for both the average and instantaneous velocities.

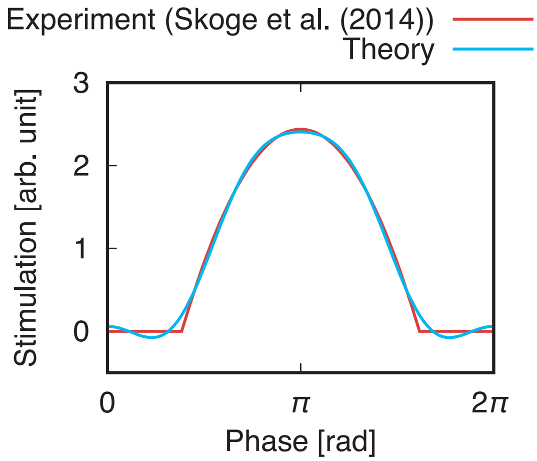

Figure S2. Forms of the stimulations S used in the experiment Ref. 8 and in the present study.

In the comparison of the average velocity between the experiment $\bar{v}_{exp}$(Ref. 8) and theory $\bar{v}_{theor}$, a distance,

$$d^{ave}(\alpha,\beta) := \sum_{T=6,10,16\text{min}} |\bar{v}_{exp}(T) - \bar{v}_{theor}(T)|, \tag{S5}$$

was calculated for each combination of parameters, α and β. Here, the average velocity was calculated from

$$\bar{v}_{theor}(T) = \frac{1}{T}\int_0^T \dot{x}_{theor}(t)dt. \tag{S6}$$

Fig. S3 shows the distance d^{ave} for various α and β. This quantity is averaged over m because the average velocity shows low dependence on m, as explained in the main text. In this calculation, the value of χ_0 is determined such that d^{ave} has the lowest value. According to the results, d^{ave} is low (less than 0.12) when $\alpha = 0.5$ and $0.4-0.8$. The dependence of the average velocity on the wave period is shown in Fig. 3 in the main text for $(\alpha,\beta) = (0.5, 0.2)$, which gives the minimum of d^{ave}.

To compare the instantaneous velocity, a distance between the experimental result $\dot{x}_{exp}$ and theoretical one $\dot{x}_{theor}$,

$$d^{inst}(m,\alpha,\beta,T,\sigma) := q\int_0^{T/q} |\dot{x}_{exp}(t) - \sigma\dot{x}_{theor}(\sigma t)|dt, \tag{S7}$$

is calculated. Here, a scaling parameter σ in the direction of t is introduced, and q denotes $\max(1,\sigma)$ because the integration is calculated in the region where both $\dot{x}_{exp}(t)$ and $\dot{x}_{theor}(\sigma t)$ are defined. As stated in the main text, σ can be physiologically interpreted as the processing speed. The value of σ is taken from $0.5 \leq \sigma \leq 2.0$ at every 0.01. With regard to χ_0, we use the value obtained by fitting to the average velocity, namely, the minimization of d^{ave}. Fig. S4 shows the distance d^{inst} for various α and β values and for a specific m minimizing d^{inst}. Fig. 2 shows the profile of the rescaled instantaneous velocity $\sigma\dot{x}_{theor}(\sigma t)$ minimizing the distance d^{inst} for $T = 6$, 10, 16 min. In the region, $T/\sigma < t < T$, the graphs are complemented by the value of $\dot{x}(T)(=\dot{x}(0))$ when $\sigma > 1$ in the figure. For reference, the distance d^{inst} when σ is fixed at 1.0 is shown in Fig. S5, and the profile of $\dot{x}_{theor}(t)$ minimizing d^{inst} is shown in Fig. S6.

According to this comparison of the instantaneous velocity, the values of parameters are estimated at $3 \leq m \leq 6$, $\alpha = 0.5$, $0.2 \leq \beta \leq 0.5$, which are consistent with the values estimated from the average velocity.

References

S1. Bellen, A. A. and Zennaro, M. Numerical methods for delay differential equations. (Oxford University Press, 2013).

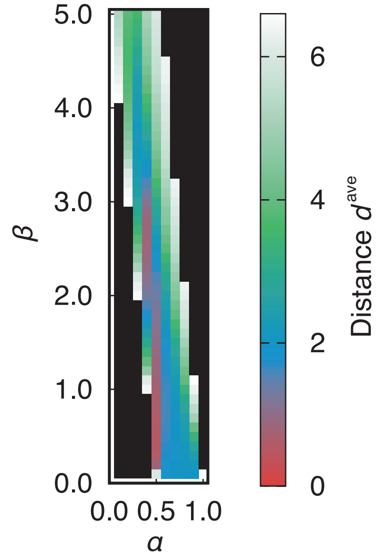

Figure S3. Distance between the average velocities obtained experimentally (Ref. 8) and theoretically, d^{ave} defined by Eq. S5, for various combination of α and β. As this quantity only minimally depends on m according to Eq. S2, the distance is averaged over m. The results are not shown in the black region because $\chi_0 < 0$ is obtained by fitting in this region.

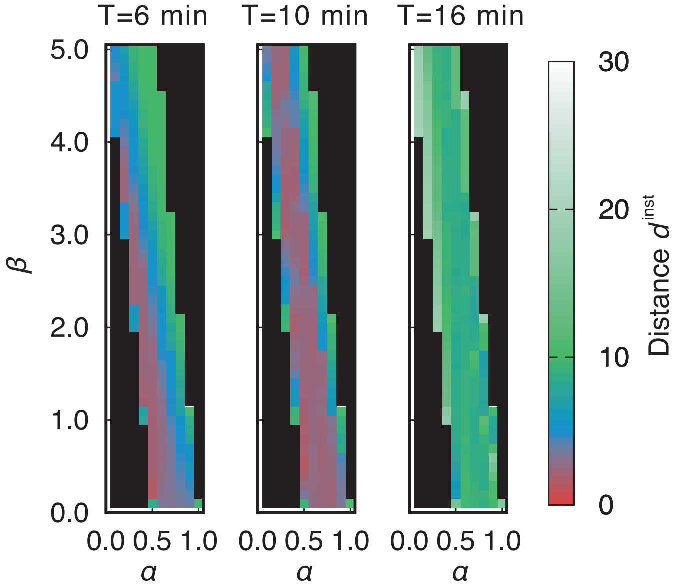

Figure S4. Distance between the instantaneous velocities obtained experimentally (Ref. 8) and theoretically, d^{inst}, defined by Eq. S7 for various combination of α and β for $T = 6$, 10 and 16 min. The results for m minimizing d^{inst} are shown. The results are not shown in the black region because $\chi_0 < 0$ is obtained by fitting in this region.

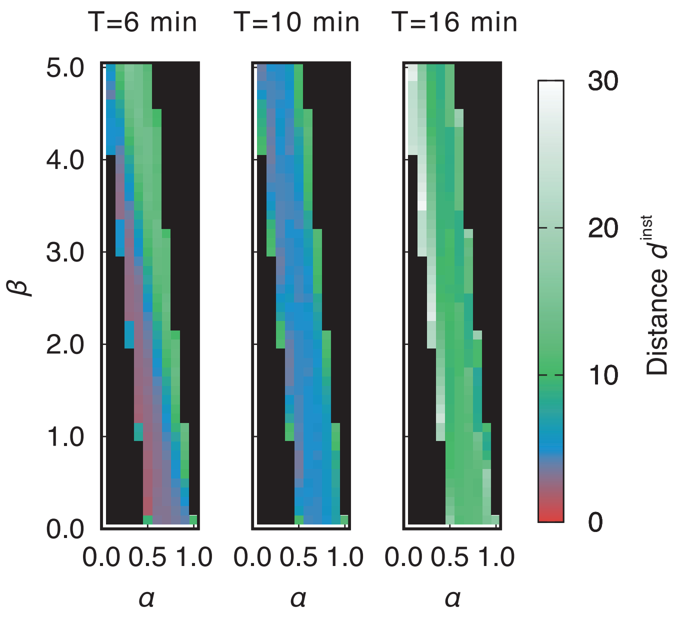

Figure S5. Distance between the instantaneous velocities obtained experimentally (Ref. 8) and theoretically, $d^{\,inst}$, defined by Eq. S7 under $\sigma = 1.0$ for various combination of α and β for T =6, 10 and 16 min. The results for m minimizing $d^{\,inst}$ are shown. The results are not shown in the black region because $\chi_0 < 0$ is obtained by fitting in this region.

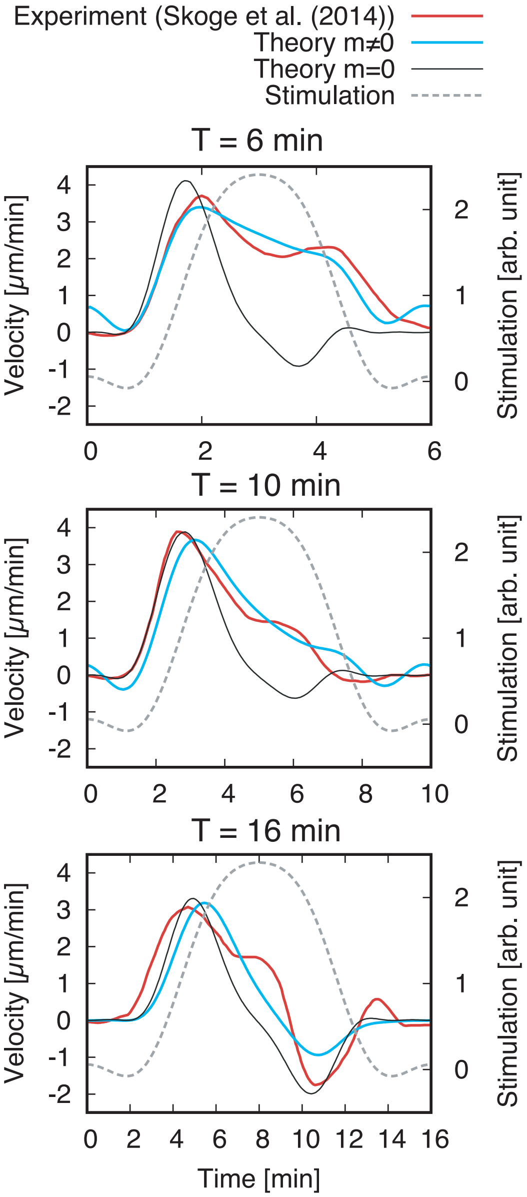

Figure S6. Instantaneous velocities when σ is fixed at 1.0. Parameter values are such that ($T = 6$ min) $m = 4$, $\alpha = 0.5$, $\beta = 0.2$ min and $\chi_0 = 1639$; ($T = 10$ min) $m = 2$, $\alpha = 0.5$, $\beta = 0.7$ min and $\chi_0 = 568$; ($T = 16$ min) $m = 1$, $\alpha = 0.8$, $\beta = 1.7$ min and $\chi_0 = 200$. For the results of $m = 0$, ($T = 6$ min) $\alpha = 0.1$, $\beta = 4.7$ min and $\chi_0 = 201$; ($T = 10$ min) $\alpha = 0.4$, $\beta = 4.6$ min and $\chi_0 = 182$; and ($T = 16$ min) $\alpha = 0.9$, $\beta = 0.9$ min and $\chi_0 = 201$. Reproduced from Skoge et al. PNAS **111**: 14448 (2014), all rights reserved.
